# Audio-Vocal Monitoring System Revealed by Mu-Rhythm Activity

**DOI:** 10.3389/fpsyg.2012.00225

**Published:** 2012-07-06

**Authors:** Takeshi Tamura, Atsuko Gunji, Hiroshige Takeichi, Hiroaki Shigemasu, Masumi Inagaki, Makiko Kaga, Michiteru Kitazaki

**Affiliations:** ^1^Department of Computer Science and Engineering, Toyohashi University of TechnologyToyohashi, Japan; ^2^Department of Developmental Disorders, National Institute of Mental Health, National Center of Neurology and PsychiatryKodaira, Tokyo, Japan; ^3^Industrial Cooperation Team, Nishina Center for Accelerator-Based ScienceRIKEN, Wako, Japan; ^4^School of Information, Kochi University of TechnologyKami, Japan

**Keywords:** event-related desynchronization, speech production, motor imagery, delayed auditory feedback, Lombard effect

## Abstract

Understanding the neural mechanisms underlying speech production has a number of potential practical applications. Speech production involves multiple feedback loops. An audio-vocal monitoring system plays an important role in speech production, based on auditory feedback about the speaker’s own voice. Here we investigated the mu-rhythm activity associated with speech production by examining event-related desynchronization and synchronization in conditions of delayed auditory feedback (DAF) and noise feedback (Lombard). In Experiment 1, we confirmed that the mu-rhythms were detectable for a conventional finger-tapping task, and vocalization. In Experiment 2, we examined the mu-rhythms for imagined speech production. We tested whether the same motor-related mu-rhythm activity was exhibited while participants listened to their own voice, and while reading. The mu-rhythms were observed for overt vocalization and covert reading, while listening to simulated auditory feedback of the participants’ own voice reading text. In addition, we found that the mu-rhythm associated with listening was boosted and attenuated under the DAF and Lombard conditions, respectively. This is consistent with the notion that auditory feedback is important for the audio-vocal monitoring system in speech production. This paradigm may help clarify the way in which auditory feedback supports motor planning, as indexed by the motor-related mu-rhythm.

## Introduction

Speech production is a complex form of motor control, requiring articulation, perception, and language processing. Previous studies have revealed that several brain areas involved in motor, auditory, and verbal processing become concurrently active in coordination during speech production (Hirano et al., 1996, 1997; McGuire et al., 1996; Paus et al., 1996). Speech production is controlled on the basis of feedback from one’s own monitored speech. In a noisy environment, the voice becomes louder and its fundamental frequency becomes higher, a phenomenon known as the Lombard effect (Lane and Tranel, 1971). Speech becomes disturbed and dysfluent in the presence of delayed feedback of the utterance i.e., under conditions of delayed auditory feedback (DAF; Lee, 1950).

These observations clearly suggest the existence of an audio-vocal monitoring system that plays an important role in speech production, on the basis of auditory feedback of one’s own voice. Levelt (1993) proposed a model incorporating internal and external feedback loops. Furthermore, Guenther et al. (2006) proposed a model in which speech production is regulated by comparing internally generated expectations before the utterance is made and sounds that are externally perceived. In this model, the dysfluency associated with the DAF conditions arises from an error in the comparison between the external perception and the internal modeling.

Generally, a motor command is accompanied by an efference copy to the sensory area where corollary discharge brought by the efference copy collates with the sensory feedback by the movements. The mechanism contributes to error detection between an expected action and the motor execution in spontaneous movements and also helps to suppress an expected sensory input. Considering the model mentioned above, replanning of the motor commands may occur if the system receives unexpected auditory feedback relative to the efference copy, which may increase repeated information processing in the motor cortex and the corollary discharge. Previous studies found that proper auditory feedback regulates motor control in speech production (e.g., Heinks-Maldonado et al., 2006).

Several neuroimaging techniques, magnetoencephalography (MEG) and functional magnetic resonance imaging (fMRI), can be used for measuring brain activation during overt and/or covert speech production (Hirano et al., 1996, 1997; Wildgruber et al., 1996; Huang et al., 2001; Sakurai et al., 2001; Blank et al., 2002). Recent developments in frequency domain analysis techniques may provide a useful tool for analyzing brain activation during speech production. Such studies have typically been focused on robust event-related desynchronization (ERD) and event-related synchronization (ERS), that is, decreases or increases of the power within a particular frequency range in electroencephalography (EEG) or MEG.

Event-related desynchronization and ERS occur contingently with body movements. ERD occurs in brain areas corresponding to the parts of the body while they are in motion, subsequently followed by ERS in mainly the alpha band (around 10 Hz) and the beta band (around 20 Hz; electrocorticography, ECoG: Crone et al., 1998a,b; surface EEG: Pfurtscheller and Lopes da Silva, 1999; surface EEG: Neuper and Pfurtscheller, 2001; MEG: Salmelin et al., 1995). An MEG study reported that the ERD and ERS around 10 Hz appeared to originate from the somatosensory cortex, while those around 20 Hz originate from the motor cortex (Caetano et al., 2007). A pattern of movement-related changes in power around 10 Hz and 20 Hz is referred to as “mu-rhythm” activity (Pfurtscheller and Neuper, 1994; Salmelin et al., 1995).

Gunji et al. (2007) used MEG to observe the ERD/ERS combination in the sensorimotor area. They have examined the MEG responses in a number of frequency bands while subjects spoke in the usual way (speaking), sang (singing), hummed (humming), and imagined (imagining) singing a popular song with normal auditory feedback in a blocked design. One trial consisted of four time stages: waiting interval (7 s), task interval (7 s), stop interval (7 s), and rest interval (10 s). Each subject performed eight trials in each condition. There were several advantages in focusing on ERD/ERS in the previous study examining the motor activity. ERD/ERS elicited by MEG is useful in identifying the cortical activities associated with a behavior, because MEG has advantages in identifying the localization of cortical sources with high spatial resolution. Also, the recording time was remarkably reduced compared with our previous studies using event related-potentials (ERP; Gunji et al., 2000, 2001). As a result, we confirmed the ERD/ERS combination of alpha (8–15 Hz), beta (15–30 Hz), and low-gamma (30–60 Hz) frequency bands during and after overt speech production (singing, speaking, and humming). The sources of the ERD/ERS combination in alpha and beta were estimated in the bilateral sensorimotor area. Also, it has been reported that imagining movement without actual movement, covert speech production, generates similar brain activation that can be reliably detected. In particular, recent reports have demonstrated that imagining movement induces patterns of ERD and ERS similar to actual movement (Pfurtscheller et al., 1997, 2006). Actually, Gunji et al. (2007) succeeded to detect ERD and ERS patterns in covert speech production similar to overt speech production.

Thus, in this study, we extended Gunji et al.’s (2007) study with EEG by investigating the cortical oscillatory changes in covert speech production and analyzed ERD/ERS changes while participants performed a task involving DAF and Lombard conditions. This would be a first report, to the authors’ knowledge, to identify ERD/ERS associated with continuous vocalization with EEG. Also, this paradigm allows us to examine whether interference to speech such as DAF and Lombard effect can be reflected by ERD/ERS in speech production.

In Experiment 1, we confirmed ERD and post-movement ERS during a conventional finger-tapping task and during overt and covert vocalization, using the frequency domain analysis of EEG. Here we also examined whether the same patterns of motor and/or auditory responses were exhibited between the overt and covert vocalization. In Experiment 2, we examined whether the same patterns of motor responses were exhibited while participants read reading (covert reading) and listened to reading. We investigated if ERD and post-movement ERS reflect an error in the comparison between unexpected external auditory feedback by DAF and Lombard effect and the internal motor plan.

## Experiment 1

We recorded EEG with (1) finger-tapping, as a body movement, (2) tongue exercise, as a similar movement to articulation, (3) articulation without vocalization, and (4) vocalization. The purpose of Experiment 1 was to examine the occurrence of ERD during speech production and post-movement ERS distributed around the sensorimotor area of the brain corresponding to the previous MEG study (Gunji et al., 2007).

### Materials and methods

#### Participants

Sixteen healthy right-handed adults (12 males and 4 females, 22–37 years old) participated in the experiment. Each participant gave written informed consent before the experiment, and was naive to the purpose of the study. The experimental protocol was approved by the committee for human subject studies at Toyohashi University of Technology (TUT), and the ethical committee at National Center of Neurology and Psychiatry (NCNP).

#### Recordings

EEG was recorded while the participants were resting and performing motor tasks. We used a Polymate AP-1000 EEG system, recording from eleven active electrodes (F8, Fz, F7, T3, C3, Cz, C4, T4, T5, Pz, and T6) placed according to the international 10–20 system, referenced to the bilateral earlobe, at a sampling frequency of 1000 Hz. Active electrodes with pre-amplifiers were used to minimize noise related to body, oral, and facial movements. The electrode impedances were below 5 kΩ although we used active electrodes. Speech sounds, videos of participants’ body movements, and electromyography (EMG) were recorded simultaneously to pick up cues about the timing of the initiation of movements. EMG was recorded from electrodes on the wrist or throat.

#### Procedure

Each trial consisted of three intervals: a 10-s long “before-movement” rest interval, a 10-s long “during-movement” activation interval, and a 10-s long “after-movement” rest interval. Thus, an individual trial lasted 30 s. After instruction on task movement of the trial, a black cross was presented at the center of the monitor. Upon presentation of the cross, the participant was asked to fixate on it, then remain motionless (the before-movement rest interval). The cross changed from black to red 10 s later, signaling initiation of the task movement. The participant performed one of five movement tasks while the cross remained red (the during-movement activation). The cross was again changed from red to black 10 s later, signaling cessation of the task movement. After the participant ceased the movement, they were required to remain motionless (the after-movement rest interval). After an “after-movement” rest interval, the black cross disappeared and the subsequent trial started with the instruction.

The five motor tasks were finger-tapping with the right hand (task 1), with the left hand (task 2), tongue exercise (task 3), articulation without vocalization (task 4), and vocalization (task 5). For the finger-tapping tasks (tasks 1 and 2), the participant placed their arm on a table and moved their index finger up and down at their own pace. We found that the average tapping speed was 2.4 taps per second, which corresponds to 24 taps in one 10 s long movement activation interval. For the tongue exercise task (task 3), the participant repeated a cycle of moving the tip of the tongue back and forth twice, left and right twice, and up and down twice. For articulation without vocalization (task 4), the participant performed articulatory movements without actual vocalization for each of all the Japanese syllables, composed of vowels or consonant-vowel pairs, exhaustively in the “alphabetical” order beginning with “a.” For the vocalization task (task 5), the participant vocalized each of all the Japanese syllables exhaustively in the same “alphabetical” order beginning with “a.” The syllables were presented at a rate of 17.5 syllables in one 10 s long movement activation interval. Tasks 1 through 5 were performed in this order, which was repeated five times. Thus there were 25 trials.

#### Analysis

Trials exceeding 120 μV in the peak-to-peak amplitude were excluded from further analyses for each recording site. A trigger signal timing the initiation of the observed movement was considered to mark the onset of the task, and was manually identified by inspection of the recorded speech sounds, video monitoring, and EMG. Hereafter, we refer to the 10-s pre-trigger interval the *pre-task* interval, the 10-s post-trigger interval the *task* interval, and the 10-s interval starting 10 s after the trigger the *post-task* interval.

A notch filter at the power line frequency was applied and no baseline correction was performed; the subsequent frequency analysis effectively eliminated the noise in the frequency bands of no interest and the baseline drift which basically consisted of a DC component. A fast Fourier transform (FFT) was performed for each interval after the artifact rejection, followed by averaging of the power spectra.

**Figure 1:**
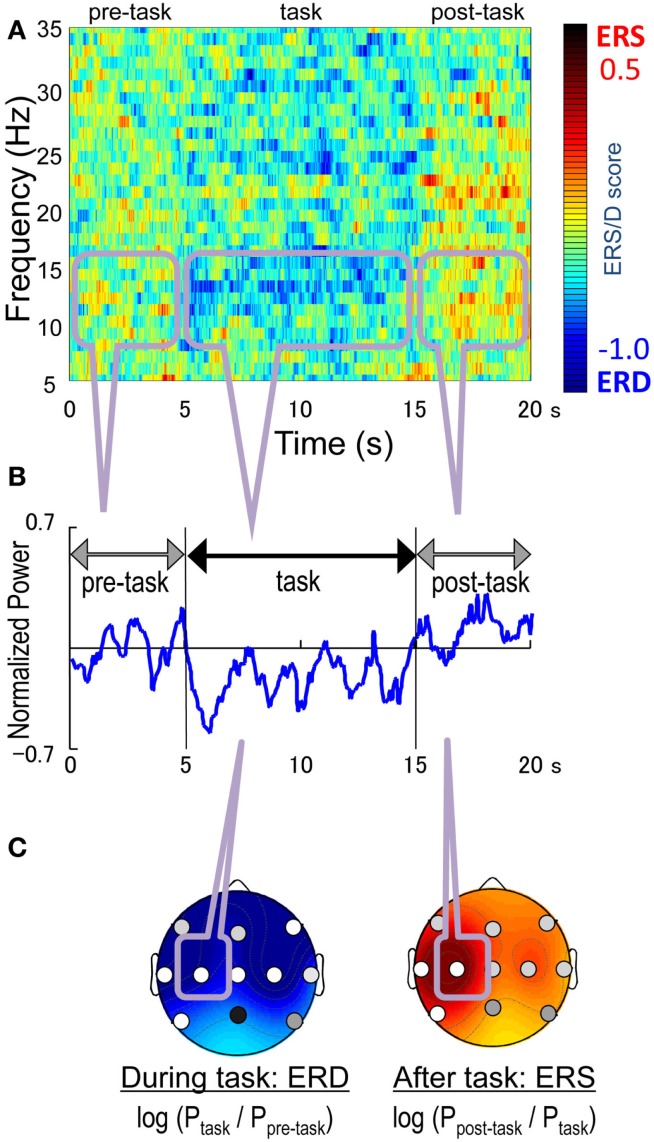
**The peak and average frequency analysis during right finger-tapping**. **(A)** the normalized power spectrum obtained by a fast Fourier transform (FFT) at C3, **(B)** an example of the averaged power of the EEG oscillation at C3 **(C)** ERD/ERS patterns. The peak frequency was the frequency with the strongest power in 8–16 Hz.

To detect the mu-rhythm arising from the sensorimotor strip, the peak frequency band was defined as a 2 Hz window centered at the frequency with the maximum power in the 8–16 Hz range during the pre-task interval (Figure 1). We then calculated the mean peak band power for the pre-task and task intervals, and their log ratio i.e., log (*P*_task-interval_) − log (*P*_pre-task-interval_) as the ERD/S score, where *P*_interval_ denotes the mean peak band power in the interval. A positive score indicated ERS, while a negative score indicated ERD during the task interval. The post-task mu-rhythm in the sensorimotor strip was detected in the same manner. The peak frequency band was defined in the same way for the post-task interval. We then calculated the post-task ERD/S score as log (*P*_post-task-interval_) −* *log (*P*_task-interval_). A positive score indicated a post-task ERS, while a negative score indicated a post-task ERD.

Statistical significance was evaluated using Student’s paired *t*-tests between the mean peak band power values during the pre-task and task intervals or the post-task and task intervals. Bonferroni correction was used for the correction for multiple comparisons.

### Results and discussion

**Figure 2:**
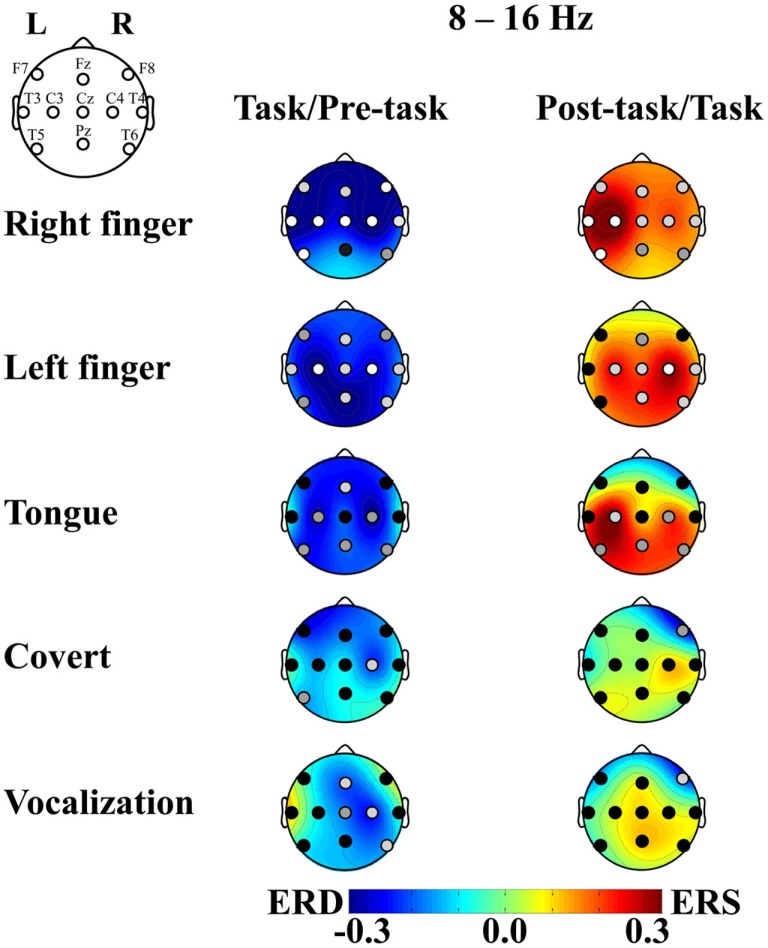
**ERD/S and *p*-value of the *t*-statistics plotted in relation to the movement tasks**. The mean across subjects is shown for (1) right finger-tapping, (2) left finger-tapping, (3) tongue exercise, (4) articulation without vocalization (abbreviated to “covert”), and (5) vocalization, from the top to the bottom. ERD is shown in cool colors while ERS is shown in warm colors. The *p*-value at each recording site is shown in the gray level of the filled circle: white, light gray, gray, and black indicate *p* < 0.001, *p* < 0.05, *p* < 0.1, and *p* > 0.1, respectively. The left column compares between the pre-task and the task intervals. The right column compares between the task and post-task intervals.

**Figure 3:**
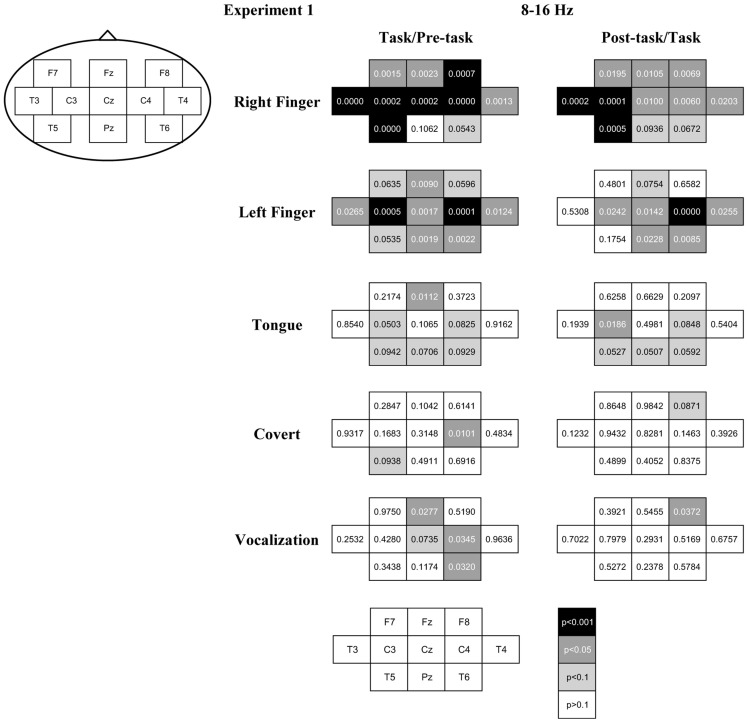
**The *p*-value of the *t*-statistics is shown at each recording site with gray scale: black, gray, light gray, and white indicate *p* < 0.001, *p* < 0.05, *p* < 0.1, and *p* > 0.1, respectively (see also Figure 2)**.

After the artifact rejection, the number of epochs for the analysis was 4.3 ± 1.6 (mean ± SD) in the right finger-tapping, 4.3 ± 1.5 in the left finger-tapping, 3.5 ± 1.9 in the tongue movements, 2.9 ± 2.1 in the articulation without vocalization, and 3.2 ± 1.9 in the speaking for individual participants. The ERD/S score for the peak band in the alpha band (8–16 Hz range; i.e., the sensorimotor response) is shown topographically in Figure 2. We observed robust ERD during the task interval and ERS during the post-task interval in the finger-tapping task. Specifically, with finger-tapping of the right hand, the ERD and the post-task ERS were observed at F7, Fz, F8, T3, C3, Cz, C4, T4, and T5 (uncorrected *p *< 0.05; with the correction for multiple comparisons, the ERD was significant at F7, Fz, F8, T3, C3, Cz, C4, T4, and T5 and the post-task ERS was significant at T3, C3, and T5, *p *< 0.05; see Figure 3). For finger-tapping of the left hand, the ERD was observed at Fz, T3, C3, Cz, C4, T4, Pz, and T6 (uncorrected *p *< 0.05; with the correction for multiple comparisons, at C3, Cz, C4, Pz, and T6, *p *< 0.05), and the post-task ERS was observed at C3, Cz, C4, T4, Pz, and T6 (uncorrected *p *< 0.05; with the correction for multiple comparisons, at C4, *p *< 0.05). In contrast, the ERD was observed only at Fz (uncorrected *p *< 0.05; n.s. with the correction for multiple comparisons), and the post-task ERS was observed only at C3 (uncorrected *p *< 0.05; n.s. with the correction for multiple comparisons) in tongue exercise. For articulation without vocalization, the ERD was observed only at C4 (uncorrected *p *< 0.05; n.s. with the correction for multiple comparisons). In the vocalization task, the significant ERD was observed at Fz, C4, and T6 (uncorrected *p *< 0.05; n.s. with the correction for multiple comparisons), and the significant post-task ERS was observed at F8 (uncorrected *p *< 0.05; n.s. with the correction for multiple comparisons). The apparent ERD and post-task ERS were observed in the finger tasks. The post-task ERS should correspond with the contralateral dominancy of the motor and somatosensory function. Orofacial movement tasks also resulted in a similar combination of ERS/ERD, though the post-task ERS did not reach statistical significance except for C3 in the tongue task, conceivably due to the shorter analyzed time-window in the orofacial movements compared with the other tasks. Weaker but significant ERD was observed for tongue movements, articulation without vocalization and for vocalization.

Global ERD during the task interval in the alpha band (8–16 Hz) was observed in the finger-tapping task. There was no statistically significant difference between the corresponding left and right sites (i.e., laterality) or between the midline and lateral sites. In contrast, the post-task ERS was prominent at T3 and C3 for right and C4 for left finger-tapping. As these recording sites correspond to the primary hand sensorimotor areas, we consider this post-task ERS to reflect an alpha mu-rhythm related to finger-tapping. Post-task ERS was stronger for the right than left finger at C3 and vice versa at C4, in accord with previous reports of cross lateralization associated with the motor control (Salmelin et al., 1995). We did not observe lateralization in the ERD during the movement interval, which may be consistent with reports of the absence of lateralization in the movement mu-rhythm in the alpha band (Salmelin et al., 1995; Ishii et al., 2002). Some other previous studies have reported cross lateralized mu-rhythm activity in the alpha band during the movement interval (Pfurtscheller et al., 1994), which may correspond to the post-task ERS we observed here.

For the tongue exercise and vocalization tasks involving movements of the phonatory organs, we observed a global ERD during the task interval, particularly at Fz. While many unresolved issues remain with respect to the control of tongue movements, the bilateral control has been consistently reported in earlier studies (e.g., Wohlert and Larson, 1991), in agreement with the prominent power change over the central regions of the motor cortex in the present study.

ERD at C4 in the right hemisphere was prominent for articulation without vocalization. ERD at Fz in midline, C4 and T6 in the right hemisphere were prominent for vocalization (Figure 3). While the oral and facial motor control including that of the tongue and throat is thought to be bilateral, Gunji et al. (2007) reported more prominent alpha band ERD in the sensorimotor cortex related to speech production in the right hemisphere during singing, compared with simple sentence utterance or humming. Right hemisphere dominance was also reported in a transcranial magnetic stimulation (TMS) study (Triggs et al., 2005). The present results might indicate right hemisphere dominance for the articulation and vocalization tasks.

**Figure 4:**
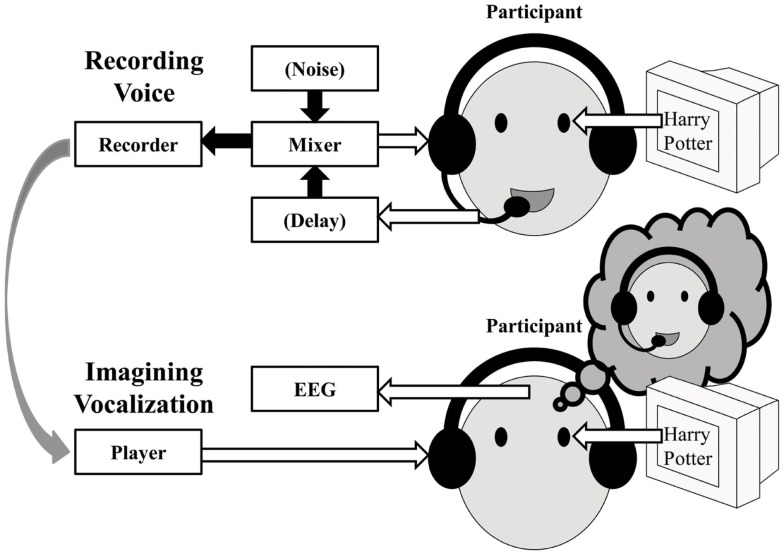
**Schematic of the procedure for Experiment 2**.

Overall, this experiment successfully demonstrated the ERD and the post-task ERS for finger-tapping (Pfurtscheller and Lopes da Silva, 1999; Neuper and Pfurtscheller, 2001). In addition, the similar pattern of ERD/ERS was also successfully detected in the tongue exercise, articulation without vocalization, and actual vocalization tasks although the power of the oscillatory change was slightly weak. A reason for the attenuated power should be that the EEG during the oral and facial movements might have been contaminated with movement artifacts, resulting in reduced ERD. Thus, we added Experiment 2 to evaluate motor-related oscillatory change in covert speech production which does not require oral and facial movements. While it is generally difficult to detect the sensorimotor mu-rhythm for actual speech production in the overt reading task due to the movement-related artifacts, Gunji et al. (2007) reported the mu-rhythm in the primary sensorimotor cortex by motor imagery alone in the covert reading task (see also Pfurtscheller et al., 1999; Hanakawa et al., 2003). Thus we expected that the mu-rhythm associated with speech production might be detected by actively generating motor imagery, and that the motor imagery with externally generated auditory information would enhance the oscillatory change by the motor imagery commands. Motor plans for speech production would be activated to generate the mu-rhythm activity if the participants listened to their own voice as simulated feedback of natural vocalization, even without actual vocalization. In addition, we might detect effects of the quality of feedback using the DAF and Lombard paradigms to investigate the relationship between the altered feedback-related mu-rhythms, which surely represents evidence of the audio-vocal monitoring system (Figure 4). Thus, we examined four feedback conditions: “normal,” “delayed,” “noise,” and “none” for a covert reading task.

## Experiment 2

The purpose of Experiment 2 was to examine occurrence of the ERD and the post-task ERS in the alpha band (8–16 Hz range) in speaking imagery alone, the “none” condition, and to identify the difference of ERD/S changes between speaking imagery with altered auditory feedback in the “normal,” “delayed,” and “noise” conditions. For the covert reading in the “delayed”-auditory feedback condition, we used the vocalization recorded during overt reading in the DAF condition as a stimulus. Because the vocalization with DAF is dysfluent and prolonged in each mora, it sounds distorted as compared to the normal recordings (Lee, 1950). The covert reading in the “noise” condition was adopted to determine if disturbance to auditory feedback affected the motor control process of vocalization.

### Materials and methods

#### Participants

Eleven participants of Experiment 1 (seven males and four females, 22–37 years old) participated in Experiment 2. They gave written informed consent before taking part in the study, and were naive to the purpose of the experiment. The experimental protocol was approved by the committee for human participant studies at TUT and the ethical committee at NCNP.

#### Recordings

The same methods and apparatus in Experiment 1 were used to record EEG while the participant was resting and performing tasks.

#### Procedure

The procedure is depicted schematically in Figure 4. There were four feedback conditions: “normal,” “delayed,” “noise,” and “none.” Before the experiment participants’ own speech sounds were recorded during overt reading as follows, which were presented as feedback stimuli during covert reading and EEG recording.

##### Sound recording

The participant read aloud sentences that were presented on a monitor. The sentences were taken from a novel (a Japanese translation of “Harry Potter”). Seven sentences were displayed on average at once for 10 s as a task interval, followed by a blank screen for 10 s as a rest interval. After the rest interval, another set of sentences was displayed for 10 s. There were three feedback conditions in the task interval: normal, delayed, and noise. In the normal feedback condition, the vocalization of the participant was auditorily fed back to him-/herself through a headset without modification. In the delayed feedback condition, the participant’s voice was fed back with a 400 ms delay using a digital signal processor. In the noise feedback condition, pink noise of 75 dB SPL was fed back. In each participant, the combination of task/rest was repeated 15 times, that is, five trials were collected in each condition. Different sentences were used for different feedback types to avoid the effects of adaptation and learning.

The recorded participants’ voice was presented as the feedback stimuli during covert reading for the subsequent EEG measurements.

##### EEG measurements in listening tasks

In the normal, delayed and noise feedback conditions, the participants listened to the corresponding one of the three recorded own voice sounds while EEG was recorded. The participants were displayed visually with the same sentences in the same manner as during the sound recording, and were instructed to covertly read the displayed sentences as they listened to their own previous readings. In the normal and delayed feedback conditions, the recorded voice was re-played. In the noise conditions, the recorded voice was re-played but mixed with the corresponding noise.

Note that listening to one’s own recorded voice is not the same as listening to one’s own voice during vocalizing. While one listens to own voice during vocalization, one does so through the air conduction and the bone conduction. In contrast, while one listens to own recorded voice, one does so only through the air conduction without the bone conduction. It makes a difference in the sound spectrum. To simulate the air-and-bone conduction under the air-only conduction, the recorded participants’ voice was re-played with attenuation by 3 dB below 1,000 Hz and boost by 3 dB above 1,000 Hz (see Shuster and Durrant, 2003). As a result, the pre-recorded voice stimuli sounded like the participants’ own real-time utterance through the headphones.

Covert reading task: In the no feedback condition, the participants were visually presented with the same sentences in the same manner as during the sound recording and instructed to read them silently without any auditory input while EEG was recorded. The no feedback condition was repeated three times using the same sentences as the normal, delayed and noise feedback conditions.

#### Analysis

The same artifact rejection was performed on the data as in Experiment 1. Data from F7 and F8 were excluded from the analyses, because recordings from these sites had to be rejected due to artifacts for most participants. The number of epochs for the analyses was 4.0 ± 1.7 (mean ± SD) in the normal condition, 4.0 ± 1.8 in the delayed feedback condition, 3.6 ± 2.0 in the noise condition, and 3.8 ± 2.0 in the no feedback condition. Frequency analysis in the 8–16 Hz range similar to Experiment 1 was performed for each of four 5 s intervals: a pre-reading 5 s interval was considered as a pre-task interval, the first 5 s as the first half task interval, the following 5 s as the second half task interval, and a 5-s post-reading interval as the post-task interval. The intervals were determined *post hoc*, and might explain a temporal influence of short term adaptation working in hearing unexpected one’s own voice of speech. The pre-task and post-task intervals were used to identify the peak frequency bands. In addition, to detect responses from the auditory cortex we evaluated the peak power in the 2–10 Hz range (see Herdman et al., 2006). The ERD/S score and the rebound ERD/S score were calculated for the auditory response in the same manner as those for the sensorimotor response.

To evaluate statistical significance, Student’s paired *t*-tests between the mean peak band power values were performed. Bonferroni correction was used for the correction for multiple comparisons. An ANOVA was performed on the non-transformed ERD/S scores, with the feedback type and the recording site as factors, for the first half task intervals, the second half task interval and the post-task interval. There were three levels of feedback type: normal, delayed and noise. The no feedback condition was not included in the ANOVA, because we used the same sentences for the with-feedback and no feedback conditions. Thus, activation in this condition may have been influenced by learning and adaptation, which could confound the results. On the other hand, since we used the same sentences in these conditions, we were able to make direct comparisons between the with-feedback and no-feedback conditions, as discussed above.

### Results and discussion

#### Sensorimotor response

**Figure 5:**
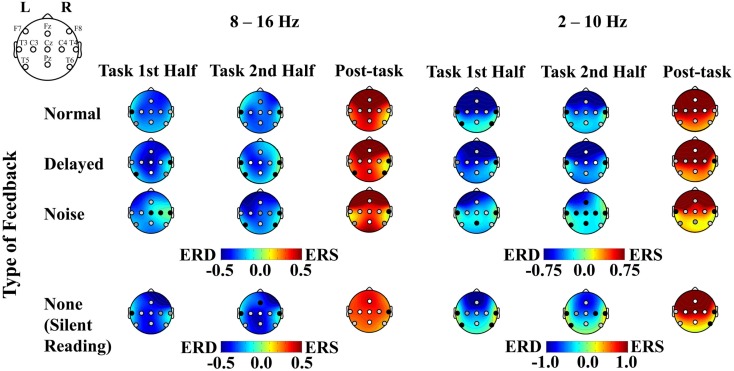
**Plots of ERD, ERS, and *p* values associated with the listening and the silent reading tasks**. ERD and ERS are shown in cool and warm colors, respectively. The left figure compares between the pre-task and the first task intervals, the middle figure compares between the pre-task and the second task intervals, and the right figure compares between the second activation and the post-task intervals. The *p*-value for each recording site is shown in white, light gray, gray, and black for *p* < 0.001, *p* < 0.05, *p* < 0.1, and *p* > 0.1, respectively. The results for the 8–16 and 2–10 Hz range are shown on the left and right sides, respectively.

**Figure 6:**
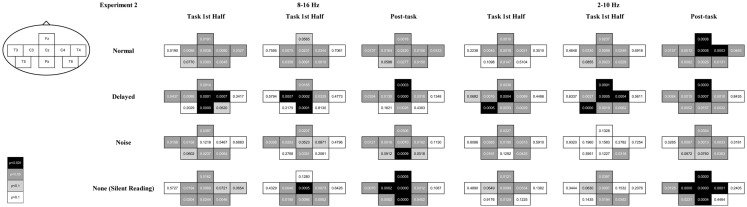
**The *p*-value of the *t*-statistics is shown at each recording site with gray scale: black, gray, light gray, and white indicate *p* < 0.001, *p* < 0.05, *p* < 0.1, and *p* > 0.1, respectively (see also Figure 5)**.

A combination of ERD during the task interval and post-task ERS was elicited in the alpha band (8–16 Hz range) over the sensorimotor strip at C3, Cz, and C4 in all the feedback conditions with a few exceptions (uncorrected *p *< 0.05; with Bonferroni correction for multiple comparisons, at Cz during the first half task interval for the normal feedback, at Cz and C4 during the first half task interval, at C3 and Cz during the second half task interval and at Cz and C4 during the post-task interval for the delayed feedback, at C3 during the post-task interval for the noise feedback, and at C3 and Cz during the second half task interval and at C3, Cz, and C4 during the post-task interval for the no feedback, *p *< 0.05; see Figures 5 and 6 for details). We successfully observed the motor-related mu-rhythm in response to the audio-visual or visual stimulation simulating speech production alone. The frequency range was almost the same as Gunji et al. (2007) reporting the mu-rhythm for vocalization (8–15 Hz). The ERD and post-task ERS we observed may indicate that the speech plans were evoked spontaneously and implicitly by auditory stimulation or covert reading.

Our results demonstrated that it was possible to observe the motor-related mu-rhythm using a realistic simulation of speech production. Thus, the mu-rhythm activity can be evoked by the audio-visual stimulation alone.

**Figure 7:**
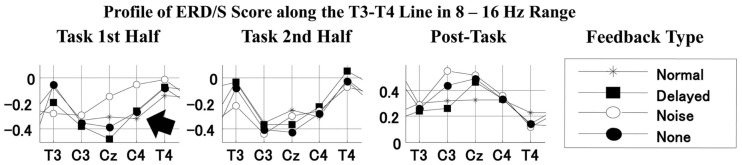
**Profiles of ERD/S score along the T3–T4 line i.e., sensorimotor strip in the 8–16 Hz range for the first task, second task, and post-task intervals for each feedback type**. Lines with stars, filled squares, open circles, and filled circles indicate the profiles for normal, delayed, noise, and no feedback (silent reading), respectively.

We also observed differential mu-rhythms depending on the auditory input. The two-way ANOVA with the feedback type and the recording site as factors showed a significant interaction for the first half task interval [*F*(16,112) = 2.268, *p *< 0.01; the corresponding *post hoc* pair-wise comparison between the noise and the other feedback types at C4 with Bonferroni correction *p *< 0.05], but not for the second half task interval [*F*(16,112) = 0.957, n.s.]. Namely, the mu-rhythm for the first half interval of the task recorded at C4 was stronger during stimulation by speech with normal or DAF than with speech with noise as shown in Figure 7. This may imply that the clarity of the simulation affects the vividness of the motor imagery as reflected in the strength of the motor-related mu-rhythm. We did not observe the same effects on the second half task interval, conceivably because of short term adaptation. The reduced mu-rhythm in the noise condition for the first half task interval may be due to incomplete acquisition of efference copy in the orofacial area of the sensorimotor cortex. Since a key to help covert reading in the noise condition was only visual cues as the sentences presented on the monitor, covert reading onset might have been delayed compared with the other tasks. Furthermore, loud noise may have distracted the participants from their covert reading. Mazard et al. (2002) reported that the performance of a mental imagery task was lower in a noisy environment than a silent environment. Thus, the developing mu-rhythm in the noise condition for the second half task interval may also indicate a temporal influence of adaptation to the loud noise. Further studies would be required to identify the temporal threshold.

Similarly, the results of the ANOVA for the post-task interval showed a significant interaction [*F*(16,112) = 2.009, *p *< 0.05]. Namely, we found a stronger post-task ERS at Fz with the stimulation by distorted than normal feedback (the corresponding *post hoc* pair-wise comparison with Bonferroni correction *p *< 0.1), conceivably because there was greater involvement of the supplementary motor area with DAF. The present results suggested that disturbance to hearing imagery of one’s own voice might seriously affect the primary stage of vocalization.

#### Auditory response

We did not observe an auditory response in the 2–10 Hz range. To confirm this lack of auditory response, we calculated the differences in the ERD/S scores between the conditions with and without feedback. Because the participants read text in both the novel stimulus (no auditory feedback) and imagined vocalization (normal, delayed, or noise feedback) conditions, the only difference between the two cases was the simulated auditory feedback. We did not find significantly greater ERS in any of the conditions with auditory feedback as compared with those without auditory feedback during the task intervals. A two-way ANOVA did not show significant effects of the feedback type [*F*(2,10) = 0.291 for the first half task interval, *F*(2,10) = 0.365 for the second half task interval, *F*(2,10) = 0.554 for the post-task interval] or any interactions between the feedback type and the recording site (*F*(16,80) = 1.125 for the first half task interval, *F*(16,80) = 0.847, or the second half task interval, *F*(16,80) = 1.013 for the post-task interval). While the lack of the auditory response to the auditory stimulation may appear odd, it should be noted that our auditory stimulus was the participants’ own voice, which is known to suppress auditory processing (Gunji et al., 2007).

Also, the lack of the auditory response might be caused by the signal-to-noise ratio of the analyzed frequency band and the number of averaged epochs should be considered. In a previous study (Herdman et al., 2006), ERS related to the simple auditory response in 2–10 Hz was obtained using an analysis time-window of maximal 50 s (250 ms × 200 epochs). However, our previous study did not obtain the ERS during overt speech vocalization using an analysis time-window of maximal 56 s (7 s × 8 epochs). Thus, the discrepancy of the results may reflect the difference of the number of analyzed epochs rather than the analyzed length. That is, we might not necessarily locate an oscillatory 2–10 Hz response in the auditory area equivalent to the N1/N100 component of ERP as an onset response to each auditory stimulus. To elucidate this problem should be a direction of further studies.

## General Discussion

In Experiment 1, we confirmed the mu-rhythms for a conventional finger-tapping task and speech production. We successfully observed the mu-rhythm activity in the alpha (8–16 Hz) band for articulation and vocalization, as well as finger-tapping. In Experiment 2, we examined the electrophysiological activity for imagined speech production. We tested whether the same pattern of the motor mu-rhythm activity was exhibited when the participants listened to their own voice and were engaged in covert reading. We successfully observed the mu-rhythms for imagined vocalizations.

We observed a central dominant ERD in the alpha band during the task interval for 8–16 Hz, in the similar range as previous reports (Hanakawa et al., 2003; Gunji et al., 2007). Interestingly, in the present study we found that the ERD, which allegedly originates in the sensorimotor cortex in the imagined vocalization for listening or covert reading, was larger in Experiment 2 than the ERD for actual vocalization in Experiment 1. While the mu-rhythm activity associated with the motor imagery is thought to be weak compared with the actual movement, it can be strengthened with training (c.f. Pantev et al., 1998; Schulz et al., 2003). Participants were expected to be trained to a greater extent with reading, than with vocalization of individual syllables. Alternatively, the pattern of activity may be related to the content of the stimuli (e.g., the amount and complexity of articulation and language). Previous studies have reported that complex articulation produces larger motor potentials originating from areas associated with speech production (Masaki et al., 1997). It remains possible that stronger motor commands are generated compared with limb movements in speech recognition and production.

In summary, the present study examined the frequency and the scalp distribution of the motor-related mu-rhythm activity for real and imagined speech production. These findings may be used as basic data in a real-time analysis of brain activation for speech production in communication such as conversation. We have also clarified that the mu-rhythms are observed not only for the overt vocalization but also for the imagined vocalization in the form of covert reading, as well as when listening to simulated auditory feedback of overt reading of sentences with normal verbal comprehension. In addition, we also revealed that the mu-rhythm for listening was boosted and attenuated with the distorted and noise feedback conditions, respectively. It is consistent with the notion that auditory feedback is important for an audio-vocal monitoring system in speech production. This paradigm may help clarify the way in which auditory feedback supports motor planning as indexed by the motor mu-rhythm.

## Conflict of Interest Statement

The authors declare that the research was conducted in the absence of any commercial or financial relationships that could be construed as a potential conflict of interest.
